# A Robust Signature Based on Autophagy-Associated LncRNAs for Predicting Prognosis in Lung Adenocarcinoma

**DOI:** 10.1155/2020/3858373

**Published:** 2020-03-02

**Authors:** Mi Zhou, Weihua Shao, Haiyun Dai, Xin Zhu

**Affiliations:** ^1^Department of Respiratory and Critical Care Medicine, The First Affiliated Hospital of Chongqing Medical University, Chongqing, China; ^2^Department of Urology, The First Affiliated Hospital of Chongqing Medical University, Chongqing, China

## Abstract

**Objective:**

To construct a predictive signature based on autophagy-associated lncRNAs for predicting prognosis in lung adenocarcinoma (LUAD). *Materials and Methods*. Differentially expressed autophagy genes (DEAGs) and differentially expressed lncRNAs (DElncRNAs) were screened between normal and LUAD samples at thresholds of ∣log_2_Fold Change∣ > 1 and *P* value < 0.05. Univariate Cox regression analysis was conducted to identify overall survival- (OS-) associated DElncRNAs. The total cohort was randomly divided into a training group (*n* = 229) and a validation group (*n* = 229) and a validation group (

**Results:**

A total of 30 DEAGs and 2997 DElncRNAs were identified between 497 LUAD tissues and 54 normal tissues; however, only 1183 DElncRNAs were related to the 30 DEAGs. A signature consisting of 13 DElncRNAs was built to predict OS in lung adenocarcinoma, and the survival analysis indicated a significant OS advantage of the low-risk group over the high-risk group in the training group, with a 5-year OS AUC of 0.854. In the validation group, survival analysis also indicated a significantly favorable OS for the low-risk group over the high-risk group, with a 5-year OS AUC of 0.737. Univariate and multivariate Cox regression analyses indicated that only positive surgical margin (vs negative surgical margin) and high-risk group (vs low-risk group) based on the predictive signature were independent risk factors predictive of overall mortality in LUAD.

**Conclusions:**

This study investigated the association between autophagy-associated lncRNAs and prognosis in LUAD and built a robust predictive signature of 13 lncRNAs to predict OS.

## 1. Introduction

Lung cancer remains a significant public health problem threatening life, with 142,670 estimated deaths in the United States in 2019 and over 1.6 million deaths worldwide annually [1, 2]. Lung cancer generally consists of small cell lung cancer (SCLC) and non-small-cell lung cancer (NSCLC), with lung adenocarcinoma (LUAD) accounting for almost 50% of NSCLC cases [3–5]. Although various therapeutic approaches have been introduced for LUAD, there were still no obvious improvements in ameliorating unfavorable prognoses, especially in patients with metastatic disease. Metastases of LUAD to the nervous system, bone, liver, adrenal gland and even urethra tend to indicate poor therapeutic outcomes, and only some selected cases may benefit from systematic therapy [6–8]. The TNM staging system provides a relatively reliable predictive model for prognosis and remains the most frequently applied predictor of survival [9]. However, a comprehensive investigation of the underlying molecular mechanisms and cellular pathways may be effective potential diagnostic tools and therapeutic targets for LUAD. Whole-exome sequencing and immune profiling analyses of LUAD indicated that molecular and immune phenotypes were associated with survival and response to adjuvant therapy in the clinical outcomes and personalized immune-based therapy of LUAD [10].

Autophagy, a highly evolutionarily conserved catabolic process, recycles and degrades cellular components via lysosomes to provide material for biomolecule synthesis [11, 12]. Malfunctions in autophagy are involved in a wide range of diseases, including cancer, neurodegeneration, and autoimmune diseases [13–16]. Autophagy is a double-edged sword with survival-supporting effects or cell death promotion in cancer cells, and it affects cancer cell responses to cytotoxic drugs [14]. Increasing evidence indicates that the interplay of autophagy and apoptosis is crucial in the pathophysiology of LUAD [17]. Long noncoding RNAs (lncRNAs), characterized by their noncoding function and their greater than 200 nucleotide length, are involved in carcinogenesis, cancer progression, and metastasis and can serve as robust diagnostic and predictive biomarkers in a variety of cancers [18–22].

Considering the significance of autophagy and lncRNAs in cancer biology, this study is aimed at investigating autophagy genes and autophagy-associated lncRNAs in LUAD from the TCGA (The Cancer Genome Atlas) database and building an effective signature based on autophagy-associated lncRNAs to predict prognosis in LUAD.

## 2. Materials and Methods

### 2.1. Data Collection

We retrieved the FPKM (fragments per kilobase of transcript per million fragments mapped) (level 3) sequencing profiles of mRNAs and lncRNAs from the TCGA data portal (https://tcga-data.nci.nih.gov/tcga/) and clinical information from the cBio Cancer Genomics Portal (http://cbioportal.org) in August 2019. The autophagy genes were collected from the Human Autophagy Database (HADb; http://www.autophagy.lu/project.html).

### 2.2. Identification of Differentially Expressed RNAs

The differentially expressed autophagy genes (DEAGs) and differentially expressed lncRNAs (DElncRNAs) were screened between LUAD and normal tissues by the “limma” package in R, with thresholds set as ∣log_2_Fold Change (FC)∣ > 1 and *P* value < 0.05. Heatmaps of the DEAGs were plotted by the “pheatmap” package.

### 2.3. Functional Enrichment Analysis of the DEAGs

Functional enrichment analysis of the DEAGs was conducted using DAVID, including biological functions, cellular components, and molecular functions, and the Kyoto Encyclopedia of Genes and Genomes (KEGG, http://www.kegg.jp/) database was searched for significant pathways (*P* < 0.05 and enrichment score > 1.5). Autophagy-related DElncRNAs (ARDElncRNAs) were identified by evaluating the expression correlations between the DElncRNAs and DEAGs using correlation scores > 0.4 in the total LUAD cohort.

### 2.4. Prognosis-Associated ARDElncRNAs

Univariate Cox regression analysis was conducted to explore overall survival- (OS-) associated ARDElncRNAs with the “survival” package in R. The interaction network between DEAGs and OS-associated ARDElncRNAs was constructed and visualized by Cytoscape v3.7.0 software.

### 2.5. Construction and Validation of the ARDElncRNA-Based Signature

The total TCGA cohort was randomly divided into a training group (*n* = 229) and validation group (*n* = 228) in a ratio of 1 : 1 by the “caret” package in R. Multivariate Cox regression analysis was performed to build a prognostic model in the training group using the “survival” package in R. The predictive value of the ARDElncRNA-based signature was evaluated by the area under the curve (AUC) values of the receiver-operator characteristic (ROC) curves in the training, validation, and total groups using the “survivalROC” package.

### 2.6. Independent Risk Factors for Overall Mortality in LUAD

The clinical variables included the age at diagnosis, sex, T status, N status, M status, AJCC TNM stage, surgical margin resection status, and risk score of the ARDElncRNA-based signature. The patients were divided by age into the following groups: ≤18-60, 60-80, >80 years old, and unknown. Sex was classified as male or female. T status was categorized as T1, T2, T3/4, and unknown. N status was negative (N0), positive (including N1, N2, and N3), and unknown. M status included negative, positive, and unknown. Stage was categorized into four types: I, II, III/IV, and unknown. Surgical margin resection status was divided into negative (R0), positive (R1/2), and unknown. The risk scores based on the ARDElncRNA-based signature were classified into low- and high-risk score groups. Univariate and multivariate Cox regression analyses were performed to explore independent predictors of OS in LUAD.

## 3. Results

### 3.1. Functional Enrichment Analysis of the DEAGs in LUAD

**Figure 1:**
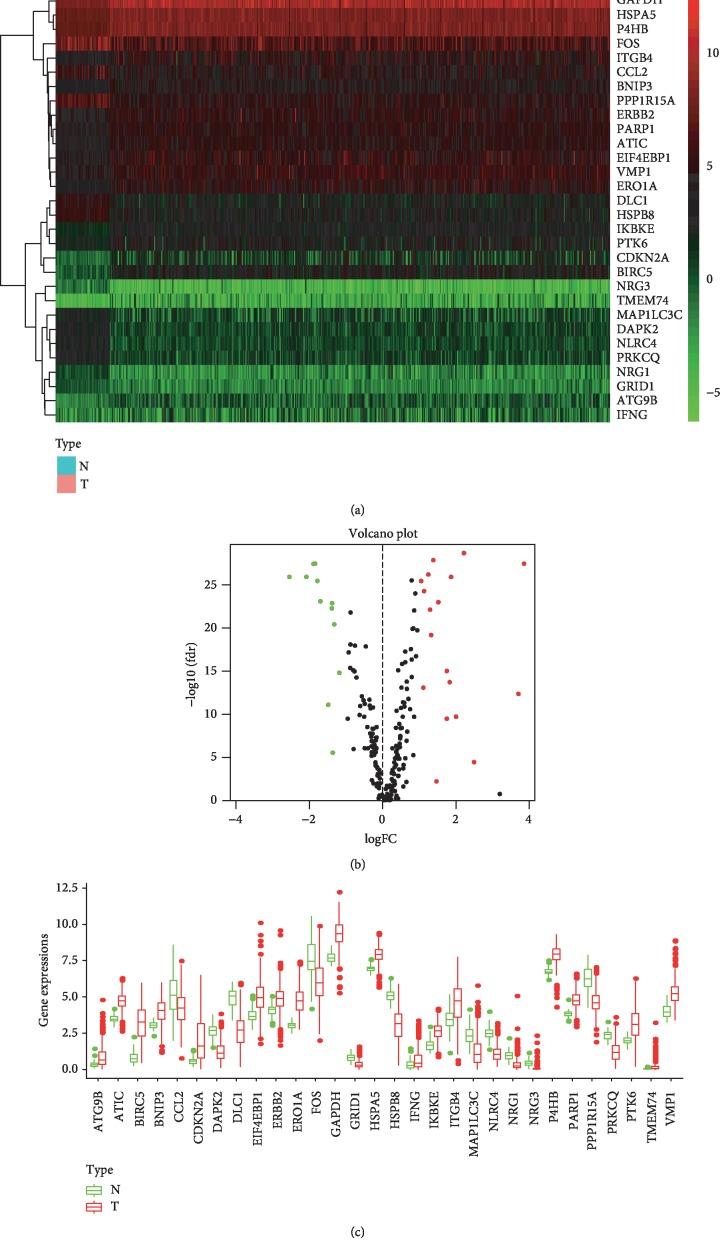
The differentially expressed autophagy genes between lung adenocarcinoma and normal tissues. (a) Heatmap of DEAGs, with red indicating high expression and green indicating low expression. (b) Volcano plot of DEAGs, with red dots indicating high expression and green dots indicating low expression. (c) Box plot of DEAGs, with the red boxes representing the tumor group and green boxes representing the normal group.

**Figure 2:**
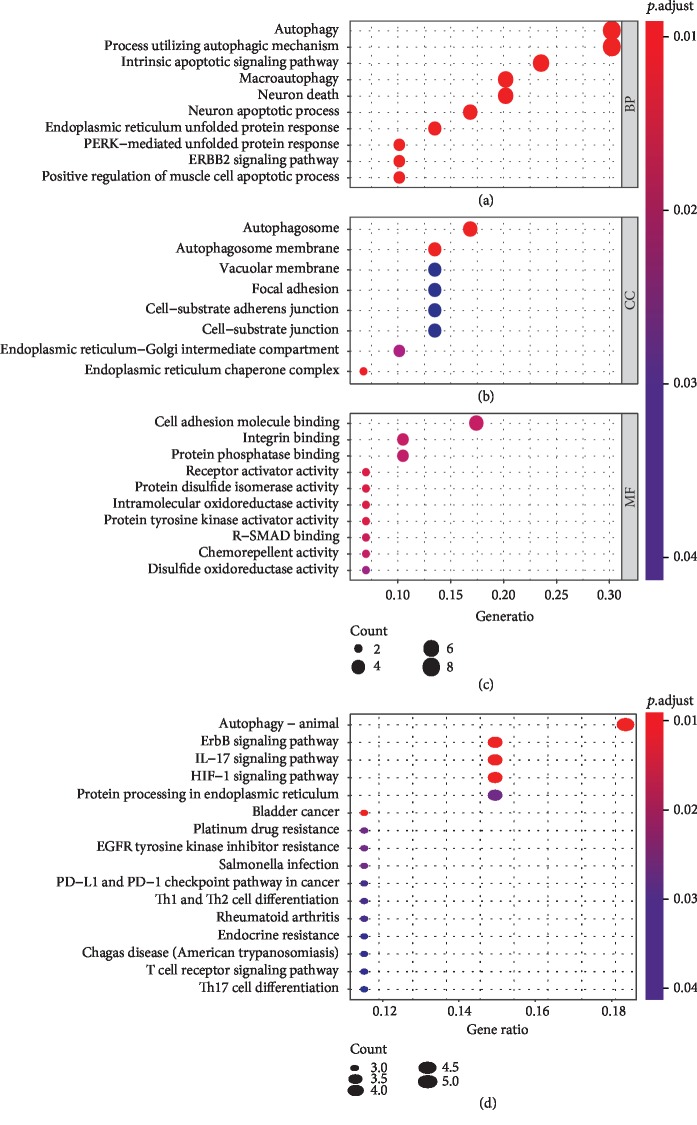
Gene functional enrichment analysis of the differentially expressed autophagy genes. (a) The top 10 biological processes in Gene Ontology analysis. (b) The top 10 cellular components in Gene Ontology analysis. (c) The top 10 molecular functions in Gene Ontology analysis. (d) The top 10 Kyoto Encyclopedia of Genes and Genomes pathways.

A total of 232 autophagy genes were collected from the HADb database, and we identified 30 DEAGs between 497 tumor tissues and 54 normal tissues from TCGA at the threshold of ∣log_2_FC∣ > 1 and *P* value < 0.05. As demonstrated in the heatmap (Figure 1(a)), volcano plot (Figure 1(b)), and boxplot (Figure 1(c)), 18 autophagy genes were upregulated, while 12 were downregulated in LUAD compared with normal tissues. Gene functional enrichment analysis indicated that autophagy-associated mechanisms were frequently implicated. “Autophagy”, “process utilizing autophagic mechanism”, and “macroautophagy” were among the top 20 biological processes; “autophagosome” and “autophagosome membrane” were the enriched cellular components; and “autophagy-animal” was the significant KEGG pathway (Figures 2(a), 2(b), and 2(d)). Moreover, we found that these DEAGs were also enriched in carcinogenesis and immunology, such as “HIF-1 signaling pathway”, “bladder cancer”, “platinum drug resistance”, “EGFR tyrosine kinase inhibitor resistance”, “PD-L1 expression and PD-1 checkpoint pathway in cancer”, “T cell receptor signaling pathway”, and “Th17 cell differentiation” in KEGG analysis (Figure 2(d)).

### 3.2. DElncRNAs in LUAD

A total of 2997 DElncRNAs were screened between normal samples and lung adenocarcinoma samples at the threshold of ∣log_2_FC∣ > 1 and *P* value < 0.05, among which 2346 DElncRNAs were upregulated while 651 DElncRNAs were downregulated. Based on the expression profiles of 497 lung adenocarcinoma tissues, 1183 ARDElncRNAs were identified with correlation scores > 0.4 and *P* value < 0.05.

### 3.3. Prognosis-Associated ARDElncRNAs in LUAD

**Figure 3:**
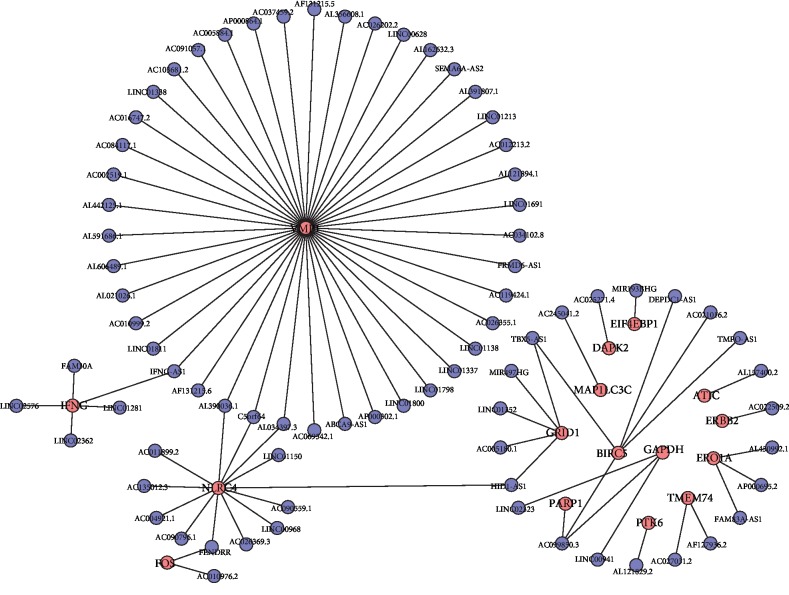
The interaction network of autophagy genes and OS-associated lncRNAs.

After the exclusion of LUAD patients with unavailable follow-up information or an OS duration less than 1 month, a total of 457 LUAD patients were included for univariate Cox regression analysis. Only 78 of 1183 ARDElncRNAs were found to be associated with the OS of LUAD patients, and expression correlations between DEAGs and OS-associated lncRNAs are summarized in Table 1. An interaction network was built on the 78 OS-associated ARDElncRNAs and 16 corresponding associated DEAGs, including ATIC, BIRC5, DAPK2, EIF4EBP1, ERBB2, ERO1A, FOS, GAPDH, GRID1, IFNG, MAP1LC3C, NLRC4, PARP1, PTK6, TMEM74, and VMP1, as shown in Figure 3.

### 3.4. OS-Associated ARDElncRNA-Based Signature for OS in LUAD

**Figure 4:**
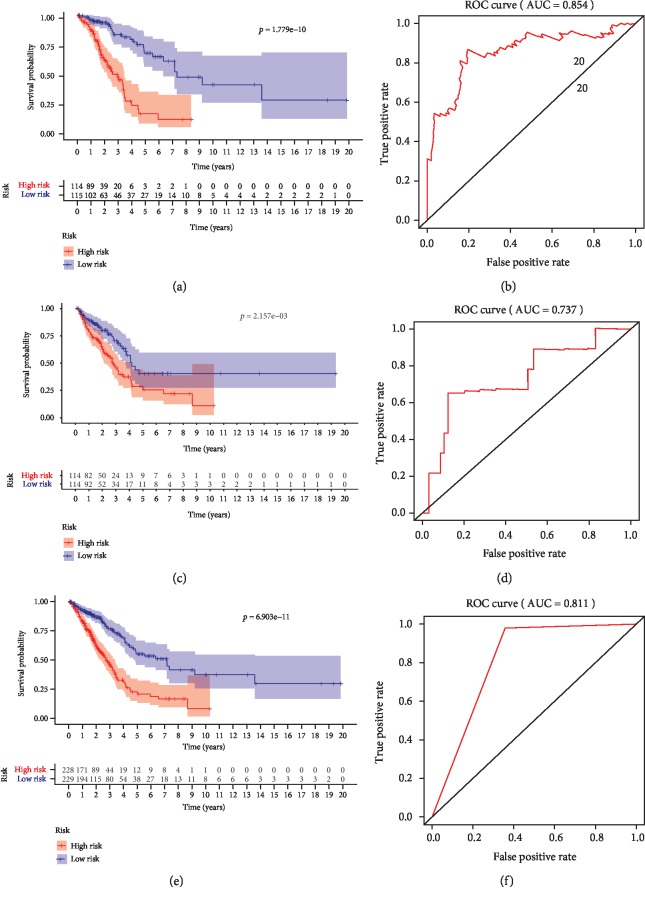
Kaplan-Meier survival curves and ROC curves to evaluate prognostic models based on the 13-lncRNA signature. (a) Kaplan-Meier survival curves for lung adenocarcinoma patients between the low- and high-risk groups in the training cohort. (b) ROC curves showing the predictive ability of the signature for 5-year OS. (c) Kaplan-Meier survival curves for lung adenocarcinoma patients between the low- and high-risk groups in the validation cohort. (d) ROC curves showing the predictive ability of the signature for 5-year OS. (e) Kaplan-Meier survival curves for lung adenocarcinoma patients between the low- and high-risk groups in the total group. (f) ROC curves showing the predictive ability of the signature for 5-year OS.

The 457 lung adenocarcinoma patients were randomly divided into a training group (*n* = 229) and a validation group (*n* = 228). The 78 identified OS-associated ARDElncRNAs were first analyzed by multivariate Cox regression in the training group to build a prognostic model. A signature consisting of 13 ARDElncRNAs was built to predict the OS of LUAD, including AC010999.2, AC084117.1, AC016747.2, LINC01338, AL356608.1, AL162632.3, SEMA6A-AS2, AC012213.2, LINC01691, AC034102.8, TMPO-AS1, AL121829.2, and AC069542.1. The detailed signature was as follows: risk score = (−4.025)^∗^AC010999.2 + (0.1964)^∗^AC084117.1 + (−1.23)^∗^ AC016747.2 + (0.4893)^∗^ LINC01338 + (−6.623)^∗^ AL356608.1 + (6.577)^∗^ AL162632.3 + (9.316)^∗^SEMA6A − AS2 + (10.78)^∗^ AC012213.2 + (1.632)^∗^ LINC01691 + (−1.12)^∗^ AC034102.8 + (0.7756)^∗^ TMPO − AS1 + (1.359)^∗^ AL121829.2 + (0.1708)^∗^ AC069542.1. The training group was further divided into a low-risk group and a high-risk group by the median risk score of the 13-ARDElncRNA signature. The Kaplan-Meier survival analysis indicated a significant OS advantage of the low-risk group over the high-risk group, as shown in Figure 4(a) (*P* < 0.001), and the sensitivity and specificity of this model in predicting OS was favorable with a 5-year OS AUC of 0.854 (Figure 4(b)).

### 3.5. Validation of the ARDElncRNA-Based Signature for OS in LUAD

**Figure 5:**
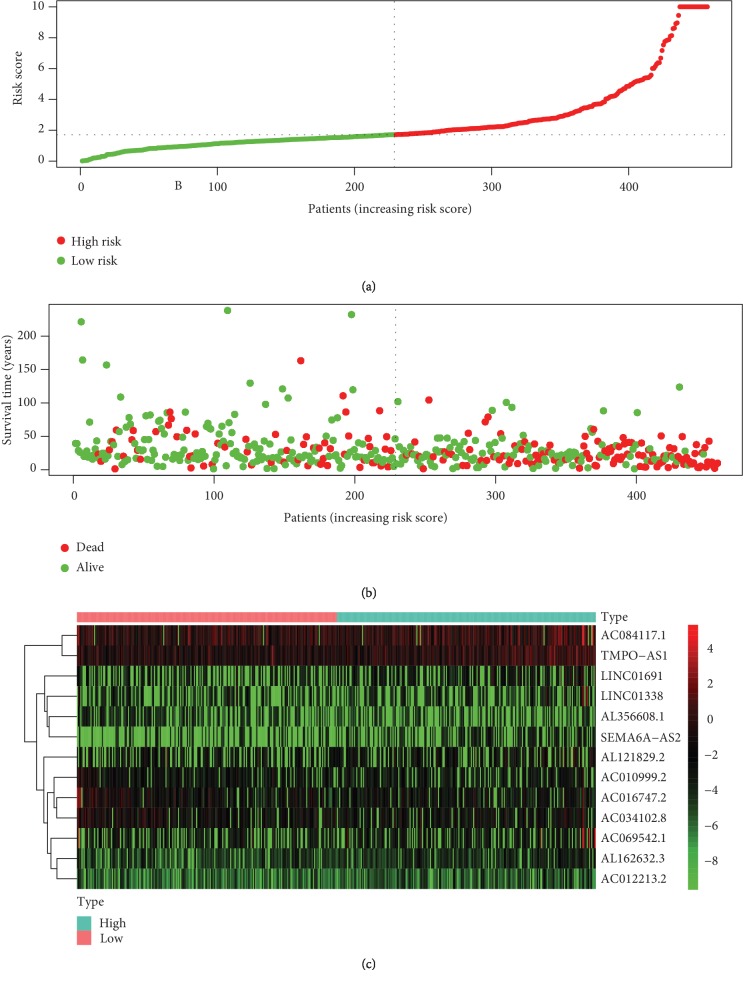
(a) The distribution of the risk scores of the 457 total included cases. (b) The distribution of OS time and status of the 457 total included cases. (c) Heatmap of the expression of the 13 lncRNAs in the low risk score and high risk score groups, with red indicating high expression and green indicating low expression.

The predictive value of the ARDElncRNA-based signature was further evaluated in the validation and total groups. The survival curve analysis indicated significantly a favorable OS for the low-risk group compared with the high-risk group (Figure 4(c), *P* < 0.001), with a 5-year OS AUC of 0.737 (Figure 4(d)) in the validation group. Moreover, in the total LUAD group combining the training and validation groups, there was still a significant difference between the low-risk and high-risk groups (Figure 4(e), *P* < 0.001), with a 5-year OS AUC of 0.811 (Figure 4(f)). The distribution of the risk scores, OS statuses, and OS times of the 457 total included patients are shown in Figures 5(a) and 5(b). The heatmap in Figure 5(c) shows the expression distributions of the 13 ARDElncRNAs in the low-risk and high-risk groups, with the color changing from green to red, indicating rising trends from low expression to high expression levels.

### 3.6. Risk Factors Predictive of Overall Mortality in LUAD

**Figure 6:**
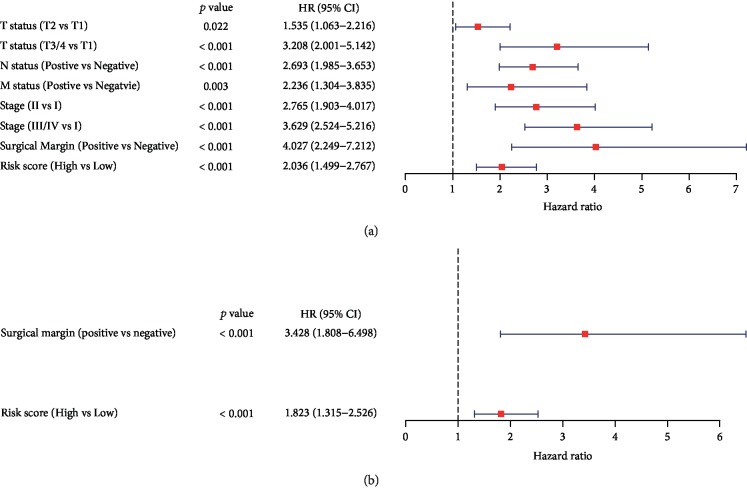
Forest plots of univariate and multivariate Cox regression analyses of risk factors associated with overall mortality in the TCGA LUAD cohort. (a) Forest plot of univariate Cox regression results. (b) Forest plot of multivariate Cox regression results.

**Figure 7:**
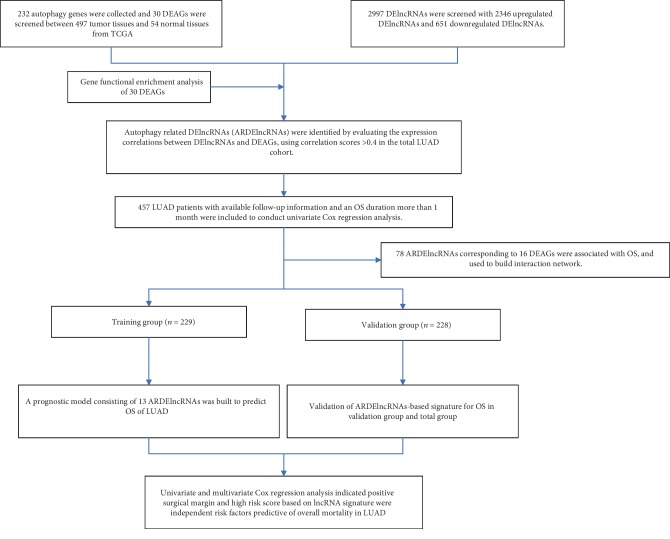
The flowchart of this study.

Univariate Cox regression analysis was conducted to investigate the influences of clinicopathological factors, including the age at diagnosis, sex, T status, N status, M status, stage, surgical margin resection status, and risk score of the ARDElncRNA-based signature. Age at diagnosis and sex were not significant factors associated with OS; however, T2 (vs T1, HR 1.535, 95% CI 1.063-2.216, *P* = 0.022), T3/4 (vs T1, HR 3.208, 95% CI 2.001-5.142, *P* < 0.001), positive N status (vs negative N status, HR 2.693, 95% CI 1.985-3.653, *P* < 0.001), positive M status (vs negative M status, HR 2.236, 95% CI 1.304-3.835, *P* = 0.003), stage II (vs stage I, HR 2.765, 95% CI 1.903-4.017, *P* < 0.001), stage III/IV (vs stage I, HR 3.629, 95% CI 2.524-5.216, *P* < 0.001), positive surgical margin (vs negative surgical margin, HR 4-027, 95% CI 2.249-7.212, *P* < 0.001), and high risk score (vs low risk score, HR 2.036, 95% CI 1.499-2.767, *P* < 0.001) were significant factors associated with OS (Table 1 and Figure 6(a)). Multivariate Cox regression analysis indicated that only positive surgical margin (vs negative surgical margin, HR 3.428, 95% CI 1.808-6.498, *P* < 0.001) and high risk score (vs low risk score, HR 1.823, 95% CI 1.315-2.526, *P* < 0.001) were independent risk factors predictive of overall mortality in LUAD (Table 2 and Figure 6(b)). The flowchart of this study is shown in Figure 7.

## 4. Discussion

Autophagy plays a dual role in suppressing and promoting initiation or progression in different phases of cancer. Autophagy was found to mediate the secretion of immune-modulating factors that promote cellular proliferation and leads to an invasive cancer phenotype [23]. The upregulation of autophagy facilitates cancer survival under stress circumstances and increases cancer growth and aggressiveness; therefore, efforts to inhibit autophagy to improve cancer therapeutic effects have attracted great interest [24].

Growing evidence indicates a close correlation between autophagy and lung cancer. PAQR3 was demonstrated to suppress the tumor progression of NSCLC cells by modulating EGFR-regulated autophagy [25]. The downregulation of autophagy facilitated the anti-LUAD efficacy of Shh pathway suppression, thus highlighting a potential approach for LUAD therapy [26]. miR-150-mediated autophagy dysfunctions were found to induce ER stress and the DNA damage response and contribute to NSCLC development [27]. With regard to cisplatin resistance in NSCLC, the autophagy inhibition of cancer stem cells identified by CD133 expression could promote the efficacy of cisplatin against NSCLC [28].

This study initially identified 30 DEAGs between 497 LUAD tissues and 54 normal tissues. Gene functional enrichment analysis revealed that the main mechanisms were involved in carcinogenesis, including bladder cancer, EGFR tyrosine kinase inhibitor resistance, and platinum drug resistance. Moreover, the immunological pathways were also suggested to correlate with the DEAGs. Targeting autophagy was found to be an alternative and novel strategy in cancer immunology. Rocaglamide, a natural product, could enhance the natural killer cell-mediated lysis of NSCLC cells by targeting ULK1, which is required for autophagy initiation and autophagy inhibition [29]. SIRP*α*D1-Fc, as a CD47-targeting fusion protein, promoted macrophage-mediated phagocytosis and cytotoxicity by inhibiting autophagy, which highlighted a potential approach for NSCLC treatment involving simultaneously targeting CD47 and autophagy [30].

Considering the increasingly significant role of lncRNAs in cancer, this study screened 2997 DElncRNAs between cancer and normal samples, and 1183 ARDElncRNAs associated with 30 DEAGs were identified. The correlations between lncRNAs and autophagy in cancer biology have been widely investigated. The upregulation of the lncRNA GAS5 was found to enhance cisplatin sensitivity in NSCLC by inhibiting autophagy [31]. The overexpression of the lncRNA NBAT1 also inhibited autophagy by interacting with PSMD10 and suppressing ATG7 transcription in NSCLC cells, which led to reduced cell viability, clonogenicity, and chemoresistance [32]. The lncRNA MSTO2P promoted lung cancer cell proliferation and autophagy by upregulating EZH2 [33]. The lncRNA BLACAT1 promoted ATG7 expression through miR-17, facilitated autophagy, and promoted the chemoresistance of NSCLC cells through the miR-17/ATG7 signaling pathway [34]. In this study, 78 OS-associated ARDElncRNAs and 16 corresponding associated DEAGs were identified and provided more molecular targets to investigate the underlying mechanism on carcinogenesis and progression of LUAD.

Taking clinical and prognostic information into consideration, 78 of 1183 ARDElncRNAs were associated with OS in LUAD. A signature consisting of 13 ARDElncRNAs was developed from the training cohort and further validated in the validation group and total group. Univariate and multivariate Cox regression analyses were conducted to investigate the influences of clinicopathological factors and the risk score of the ARDElncRNA-based signature, and only positive surgical margin (vs negative surgical margin) and high score (vs low score) based on the ARDElncRNA-based signature were independent risk factors predictive of overall mortality in LUAD.

However, there were some limitations in our study. First, the ARDElncRNAs were identified based on the expression correlations between DElncRNAs and DEAGs. The underlying mechanisms and molecular correlations between ARDElncRNAs and autophagy need to be investigated. Moreover, the ARDElncRNA signature was developed and validated using a retrospective cohort from TCGA, and the predictive efficacy needs to be further proven in other prospective cohorts.

## 5. Conclusions

This study first investigated the correlation between autophagy-associated lncRNAs and prognosis in LUAD and built a robust predictive signature of 13 ARDElncRNAs to predict OS.

## Figures and Tables

**Table 1:** Expression correlations between DEAGs and OS-associated lncRNAs.

DEAGs	DElncRNAs	Correlation	*P* values
ATIC	AL157400.2	0.444360577	1.82*E*-25
BIRC5	DEPDC1-AS1	0.4832965	1.88*E*-30
BIRC5	AC021016.2	-0.433811504	3.18*E*-24
BIRC5	TMPO-AS1	0.567742456	9.45*E*-44
BIRC5	AC099850.3	0.730138322	6.65*E*-84
BIRC5	TBX5-AS1	-0.436332494	1.62*E*-24
DAPK2	AC025271.4	0.416487849	2.84*E*-22
EIF4EBP1	MIR193BHG	0.40665219	3.24*E*-21
ERBB2	AC022509.2	0.453023068	1.60*E*-26
ERO1A	AL450992.1	0.459664478	2.37*E*-27
ERO1A	AP000695.2	0.423841103	4.36*E*-23
ERO1A	FAM83A-AS1	0.404049204	6.10*E*-21
FOS	FENDRR	0.401599724	1.10*E*-20
FOS	AC010976.2	0.415847677	3.34*E*-22
GAPDH	LINC00941	0.474791308	2.62*E*-29
GAPDH	AC099850.3	0.41089708	1.15*E*-21
GAPDH	LINC02323	0.428872623	1.18*E*-23
GRID1	HID1-AS1	0.410196674	1.36*E*-21
GRID1	AC005180.1	0.424510968	3.67*E*-23
GRID1	LINC01352	0.479484409	6.17*E*-30
GRID1	MIR497HG	0.406012066	3.79*E*-21
GRID1	TBX5-AS1	0.519536812	1.05*E*-35
IFNG	LINC02576	0.502638658	3.54*E*-33
IFNG	IFNG-AS1	0.509396601	3.59*E*-34
IFNG	FAM30A	0.420786534	9.55*E*-23
IFNG	LINC01281	0.46391669	6.84*E*-28
IFNG	LINC02362	0.520330327	7.92*E*-36
MAP1LC3C	AC245041.2	0.402043463	9.88*E*-21
NLRC4	AC090796.1	0.427690851	1.60*E*-23
NLRC4	HID1-AS1	0.406839642	3.10*E*-21
NLRC4	AC004921.1	0.520206459	8.28*E*-36
NLRC4	AC135012.3	0.422379934	6.35*E*-23
NLRC4	AL034397.3	0.615024927	4.82*E*-53
NLRC4	C5orf64	0.545917326	6.02*E*-40
NLRC4	AC011899.2	0.700536094	1.49*E*-74
NLRC4	LINC01150	0.521684592	4.89*E*-36
NLRC4	AC090559.1	0.649814293	5.88*E*-61
NLRC4	LINC00968	0.451512303	2.46*E*-26
NLRC4	AC026369.3	0.524436025	1.82*E*-36
NLRC4	AL390036.1	0.62122729	2.21*E*-54
NLRC4	FENDRR	0.412286149	8.12*E*-22
PARP1	AC099850.3	0.427391558	1.73*E*-23
PTK6	AL121829.2	0.430579374	7.51*E*-24
TMEM74	AF127936.2	0.481028722	3.82*E*-30
TMEM74	AC027031.2	0.566844781	1.37*E*-43
VMP1	AF131215.6	0.408834647	1.90*E*-21
VMP1	IFNG-AS1	0.604256223	8.65*E*-51
VMP1	LINC01811	0.480470855	4.54*E*-30
VMP1	AC010999.2	0.64087377	7.96*E*-59
VMP1	AL021026.1	0.515098693	4.99*E*-35
VMP1	AL606489.1	0.453317152	1.47*E*-26
VMP1	AL034397.3	0.410714008	1.20*E*-21
VMP1	AL591686.1	0.43870005	8.55*E*-25
VMP1	AL442125.1	0.438302333	9.52*E*-25
VMP1	AC002519.1	0.678129757	3.29*E*-68
VMP1	AC084117.1	0.724641884	4.48*E*-82
VMP1	AC016747.2	0.506683107	9.04*E*-34
VMP1	C5orf64	0.544372157	1.09*E*-39
VMP1	LINC01338	0.459058135	2.83*E*-27
VMP1	AC103681.2	0.51428241	6.64*E*-35
VMP1	AC091057.1	0.51146999	1.76*E*-34
VMP1	AC005884.1	0.605423729	4.97*E*-51
VMP1	AP000864.1	0.519643474	1.01*E*-35
VMP1	AC037459.2	0.632197563	8.00*E*-57
VMP1	AF131215.5	0.456755168	5.51*E*-27
VMP1	AL356608.1	0.519080165	1.23*E*-35
VMP1	AC026202.2	0.756218065	3.10*E*-93
VMP1	LINC00628	0.446931586	8.90*E*-26
VMP1	AL162632.3	0.508247027	5.31*E*-34
VMP1	SEMA6A-AS2	0.408303227	2.17*E*-21
VMP1	AL391807.1	0.482667739	2.29*E*-30
VMP1	LINC01213	0.616028773	2.94*E*-53
VMP1	AC012213.2	0.554034976	2.50*E*-41
VMP1	AL390036.1	0.468472261	1.77*E*-28
VMP1	AL121894.1	0.430161293	8.38*E*-24
VMP1	LINC01691	0.423537637	4.71*E*-23
VMP1	AC034102.8	0.687069659	1.14*E*-70
VMP1	FRMD6-AS1	0.533454663	6.76*E*-38
VMP1	AC119424.1	0.406469336	3.39*E*-21
VMP1	AC026355.1	0.675391559	1.80*E*-67
VMP1	LINC01138	0.511085231	2.01*E*-34
VMP1	LINC01337	0.442013564	3.47*E*-25
VMP1	LINC01798	0.469674869	1.23*E*-28
VMP1	LINC01800	0.519909371	9.19*E*-36
VMP1	AP000302.1	0.571330031	2.10*E*-44
VMP1	ABCA9-AS1	0.438916673	8.06*E*-25
VMP1	AC069542.1	0.411642121	9.52*E*-22

**Table 2:** Univariate and multivariate Cox regression analyses of LUAD.

Variables	Total (*n* = 457)	Univariate Cox regression			Multivariate Cox regression		
		HR	95% CI	*P*	HR	95% CI	*P*
Age (years)							
18–60	130	**1**	Reference	NA	**1**	Reference	NA
60–80	292	**1.124**	**0.800-1.580**	**0.5**	NA	NA	NA
>80	25	**1.338**	**0.653-2.740**	**0.426**	NA	NA	NA
Unknown	10	NA	NA	NA	NA	NA	NA
Sex							
Male	249	**1**	Reference	NA	**1**	Reference	NA
Female	208	**1.083**	**0.802-1.461**	**0.603**	NA	NA	NA
T status							
T1	155	**1**	Reference	NA	**1**	Reference	NA
T2	244	**1.535**	**1.063-2.216**	**0.022**	**1.149**	**0.780-1.691**	**0.483**
T3/4	55	**3.208**	**2.001-5.142**	**<0.001**	**1.447**	**0.820-2.553**	**0.202**
Unknown	3	NA	NA	NA	NA	NA	NA
N status							
Negative	295	**1**	Reference	NA	**1**	Reference	NA
Positive	151	**2.693**	**1.985-3.653**	**<0.001**	**1.551**	**0.911-2.641**	**0.106**
Unknown	11	NA	NA	NA	NA	NA	NA
M status							
Negative	307	**1**	Reference	NA	**1**	Reference	NA
Positive	23	2.236	1.304-3.835	0.003	0.948	0.485-1.854	0.877
Unknown	127	NA	NA	NA	NA	NA	NA
Stage							
Stage I	247	**1**	Reference	NA	**1**	Reference	NA
Stage II	104	**2.765**	1.903-4.017	**<0.001**	**1.69**	0.940-3.039	0.08
Stage III/IV	98	3.629	2.524-5.216	**<0.001**	1.798	0.886-3.649	0.104
Unknown	8	NA	NA	NA	NA	NA	NA
Surgical margin							
Negative (R0)	310	**1**	Reference	NA	**1**	Reference	NA
Positive (R1/2)	16	4.027	2.249-7.212	**<0.001**	3.428	1.808-6.498	**<0.001**
Unknown	131	NA	NA	NA	NA	NA	NA
Risk score							
Low	229	**1**	Reference	NA	**1**	Reference	NA
High	228	2.036	1.499-2.767	**<0.001**	1.823	1.315-2.526	**<0.001**

## Data Availability

The authors declare that the data supporting the findings of this study is available within the article.

## Abbreviations

TCGA: The Cancer Genome Atlas
DEAGs: Differentially expressed autophagy genes
DElncRNAs: Differentially expressed lncRNAs
OS: Overall survival
AUC: Areas under curve
ROC: Receiver operatoring characteristic
SCLC: Small cell lung cancer
NSCLC: Non-small-cell lung cancer
LUAD: Lung adenocarcinoma
KEGG: Kyoto Encyclopedia of Genes and Genomes
ARDElncRNAs: Autophagy-related DElncRNAs
FC: Fold change.
